# Hydrocephalus as the presenting symptom of sarcoidosis: A case report and review of literature

**DOI:** 10.1002/ccr3.2665

**Published:** 2020-01-14

**Authors:** Rachel J. Saban, Meaghan M. Berns, Mazen M. Al‐Hakim, Gustavo A. Patino

**Affiliations:** ^1^ Oakland University William Beaumont School of Medicine Rochester MI; ^2^ Department of Neurology Beaumont Hospital Royal Oak MI; ^3^ Department of Foundational Medical Studies Oakland University William Beaumont School of Medicine Rochester MI

**Keywords:** granulomatous disease, hydrocephalus, neurosarcoidosis, sarcoidosis

## Abstract

Hydrocephalus is rare in sarcoidosis, especially as the presenting symptom. Neurosarcoidosis as a cause of unexplained communicating hydrocephalus should be considered in cases of abnormal cerebrospinal fluid (CSF) and negative infectious and tumoral studies.

## Graphical abstract

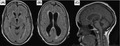

## 1 INTRODUCTION

Sarcoidosis is a granulomatous disorder of unknown origin that can affect multiple organ systems. Most commonly, it affects the lungs, skin, and lymphatic system. It typically presents in young adults. Central nervous system (CNS) involvement occurs in only 5%‐10% of cases and increases morbidity and mortality significantly.^1^ The most common neurologic finding in neurosarcoidosis is cranial neuropathies, predominantly peripheral facial palsy (occurring in 50% of patients), and optic neuritis followed by palate dysfunction, hearing abnormalities, and vertigo.^2^, ^3^, ^4^ Other manifestations include hypothalamic and pituitary abnormalities, chronic aseptic meningitis, mass‐lesion effect, and seizures.^1^ Hydrocephalus occurs in only 6% of cases of neurosarcoidosis, but of all manifestations of CNS involvement, it has been reported to confer the worst long‐term prognosis.^5^, ^6^, ^7^

In most reported cases of neurosarcoidosis with hydrocephalus, the patient had a previous diagnosis of systemic sarcoidosis. Hydrocephalus as the presenting symptom of sarcoidosis in a previously healthy patient is an atypical presentation that is exceedingly rare and challenging to diagnose.^1^, ^6^ We present the case of a previously healthy 52‐year‐old woman with significant hydrocephalus of all four ventricles as the presenting manifestation of sarcoidosis.

## 2 CASE PRESENTATION

**Figure 1:**
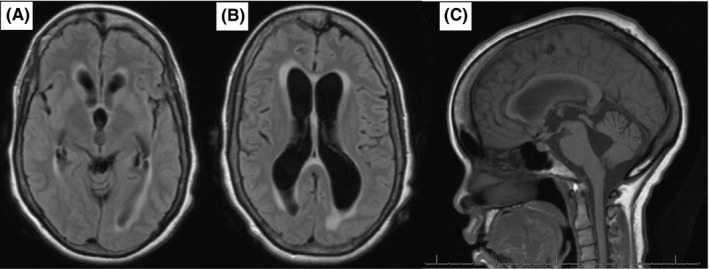
Dilation of third and lateral ventricles with FLAIR sequence (A, B). Enlargement of the third and fourth ventricles with patent cerebral aqueduct on T1 sagittal sequence (C)

**Figure 2:**
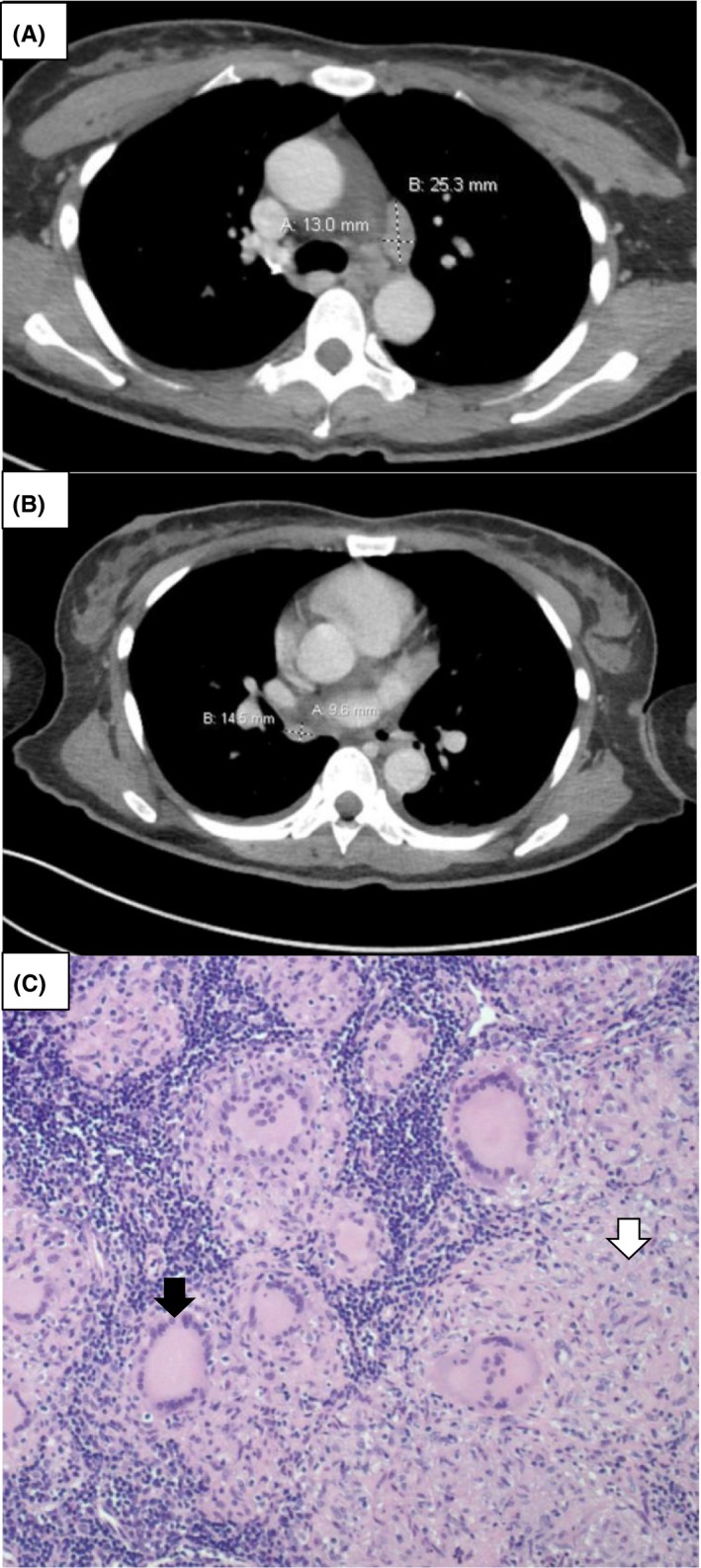
Chest CT demonstrating bilateral enlarged thoracic lymph nodes of 13.0 X 25.3 mm and 9.6 X 14.5 mm consistent with sarcoidosis (A, B). Lymph node biopsy demonstrating granulomatous inflammatory reaction with multinucleate giant cells (black arrowhead) and fibrosis (white arrowhead) (C)

A 53‐year‐old African‐American woman with no past medical history visited the emergency department due to progressive nausea and vomiting. She had a month‐long course of fatigue, headaches, intermittent vertigo, tinnitus, and ataxia. Review of systems revealed she had an unintentional weight loss of 30 pounds over the last 4 months. Neurologic examination showed right gaze‐evoked nystagmus, postural instability, and an unsteady shuffling gait. Fundoscopic examination showed no papilledema or other abnormalities. Respiratory examination was normal. Her complete blood count and routine biochemistry were normal. C‐reactive protein and erythrocyte sedimentation rate (ESR) were within normal limits. Serum level of angiotensin‐converting enzyme (ACE) was 51 U/L (normal range: 12‐60 U/L). Brain computed tomography (CT) revealed remarkable hydrocephalus with dilation of all four ventricles. Magnetic resonance imaging (MRI) showed patency of the cerebral aqueduct and ruled out cerebral or subarachnoid mass lesions, consistent with communicating hydrocephalus (Figure 1). Lumbar puncture was performed in light of clinical and radiographic findings. Cerebrospinal fluid (CSF) was transparent and showed lymphodominant pleocytosis with increased protein level (104 mg/dL), markedly decreased glucose (21 mg/dL), and normal opening pressure. Chest CT showed bilateral lymphadenopathy, and biopsy revealed noncaseating granulomas admixed with giant cells without evidence of malignancy or infection (Figure 2). A diagnosis of neurosarcoidosis with hydrocephalus was made, and the patient was started on 1 g of intravenous solumedrol for 5 days with a subsequent oral prednisone taper with methotrexate. Three months later, she received a ventricular shunt for persistent severe gait disturbance. She returned to walking independently within one month of the procedure and has since shown marked improvement in all neurologic symptoms.

## 3 DISCUSSION

Hydrocephalus is a very rare complication of sarcoidosis, especially as the presenting symptom.^6^ We present one such case in this report. To the best of our knowledge, only 21 others exist in the literature, of which we were able to include 19 in this report (Table 1).^7^, ^8^, ^9^, ^10^, ^11^, ^12^, ^13^, ^14^, ^15^, ^16^, ^17^, ^18^, ^19^, ^20^, ^21^, ^22^ Hydrocephalus typically presents with nonspecific symptoms, and the diagnosis is made based on imaging.^8^ The differential diagnosis is extensive (Table 2) making the correct attribution difficult due to a lack of definitive diagnostic testing for neurosarcoidosis. As such, a high degree of clinical suspicion must be maintained.

**Table 1:** Hydrocephalus as the presenting symptom in systemic sarcoidosis cases reported in the literature, including the current case

Author	ESR/CRP	Serum ACE	CSF Findings	Diagnosis	Type	Treatment	Outcome
This report	ESR: Normal, CRP: Normal	Normal	Lymphodominant pleocytosis, elevated protein, hypoglycorrhachia	Lymphadenopathy on chest CT	Communicating	Corticosteroids + methotrexate, followed by VP months later	Partial recovery
Brouwer 2009^9^		Normal	Lymphodominant pleocytosis, elevated protein	Lymphadenopathy on FDG‐PET	Communicating	Corticosteroids	Complete recovery
Muayqil 2006^16^	ESR: Normal		Lymphodominant pleocytosis	Lymphadenopathy on CXR and chest CT; meningeal and hypothalamic enhancement in brain MRI	Communicating	VP shunt + corticosteroids	Partial recovery
Muniesa 2006^17^		Elevated	Leukocytic pleocytosis	Cutaneous lesions	Communicating	VP shunt + corticosteroids	Complete recovery
Onoda 2004			Lymphodominant pleocytosis, elevated protein, hypoglycorrhachia	Increased ACE in CSF	Communicating	VP shunt + corticosteroids	Died from nosocomial pneumonia
Sano 2015^18^			Lymphodominant pleocytosis, protein elevated protein, hypoglycorrhachia, ACE normal	Lymphadenopathy on chest CT; meningeal lesions in basal cisterns	Communicating	VP shunt + corticosteroids +Methotrexate + Infliximab	Partial recovery
Sugiyama 2016^19^	ESR: Elevated, CRP: Normal	Normal	Leukocytic pleocytosis, elevated protein	Lymphadenopathy on whole body contrast CT and FDG‐PET	Communicating	VP shunt + corticosteroids	Partial recovery
Zoja 2012^21^				Autopsy	Communicating		Death
Benzagmout 2007^8^		Elevated	Elevated opening pressure, lymphodominant pleocytosis, elevated protein, hypoglycorrhachia	Cervical and submandibular lymphadenopathy	Noncommunicating	External Ventricular Drain + Corticosteroids	Partial recovery
Berhouma 2009^23^				Brain MRI with temporal trapped horn and multiple enhancing lesions in subarachnoid space	Noncommunicating	Right temporal tip lobectomy + corticosteroids	Complete recovery
Brouwer 2009^9^		Normal	Lymphodominant pleocytosis, elevated protein	Lymphadenopathy on FDG‐PET	Noncommunicating	Ventriculoscopy assisted fenestration of lateral ventricle cyst	Complete recovery
Chandna 2015^10^		Normal		Lymphadenopathy on CXR	Noncommunicating	VP shunt + corticosteroids	Death
Chiang 2002^11^		Elevated		Cutaneous lesions	Noncommunicating	VP shunt + corticosteroids	
Hitti 2015^12^			Normal	Leptomeningeal enhancement in brain and spine MRI months later	Noncommunicating	VP shunt + corticosteroids +Mycophenolate mofetil	
Kim 2012^13^			Leukocytic pleocytosis, elevated protein	Lymphadenopathy on CXR	Noncommunicating	VP shunt + corticosteroids	Complete recovery
Matsuda 2015^14^		Normal	Lymphodominant pleocytosis, hypoglycorrhachia	Neuroendoscopic biopsy of enhancing ventricular lesions	Noncommunicating	Ventriculostomy, followed by VP shunt + corticosteroids	Complete recovery
McKeever 2019^15^				Nodular lesions in brain MRI years later	Noncommunicating	Endoscopic third ventriculostomy, years later recurred and required shunt	Complete recovery first episode
Tabuchi 2013^24^	ESR: Elevated, CRP: Normal	Normal	Pleocytosis, elevated protein	Lymphadenopathy on CXR	Noncommunicating	VP shunt + corticosteroids	Partial recovery
Westhout 2008^20^	ESR: Elevated		Elevated protein	Biopsy of temporal lobe lesion	Noncommunicating	VP shunt + corticosteroids	Complete recovery
Yoshitomi 2015^25^	CRP: Elevated		Elevated opening pressure, hypoglycorrhachia	Diffuse leptomeningeal enhancement in brain MRI and mass lesions in third and fourth ventricles	Noncommunicating	Endoscopic fenestration foramen of Magendie, followed by VP shunt + corticosteroids	Complete recovery

**Table 2:** Differential diagnosis of acquired hydrocephalus in adults

Subarachnoid/Intraventricular Hemorrhage
Trauma
Tumor and metastases (including of the leptomeninges)
Meningitis/encephalitis
Bacterial (including syphilis)
Viral (including EBV and HIV)
Fungal
Infectious etiologies
Tuberculosis
Lyme disease
Toxoplasmosis
Neurocysticercosis
Whipple's disease
Inflammatory etiologies
Sarcoidosis
Systemic lupus erythematosus (SLE)
Behçet's disease
Wegner's granulomatosis

Hydrocephalus secondary to sarcoidosis can present as communicating or noncommunicating. The latter scenario is the most common (60% of cases included in this report), and the presence of mass lesions in the CNS can raise the possibility of the diagnosis and even provide a site for biopsy. In the case of communicating hydrocephalus, when the differential diagnosis includes infectious or neoplastic pathologies of the leptomeninges, additional studies (including CSF analysis and chest imaging) usually provide the clues for diagnosis. In 50% of the patients, the diagnosis was suspected when lymphadenopathy was found, most commonly in chest imaging. Among patients with communicating hydrocephalus, this was the most common way to make the diagnosis (63%), including in our patient. In two of those patients, there were also abnormal findings in the contrast MRI (Table 1). In one patient, the diagnosis was made by biopsy of cutaneous lesions, in another, it was suggested by elevated ACE levels in CSF, and in the last case, the patient died suddenly from acute hydrocephalus and the diagnosis was made by pathological examination of the leptomeninges at autopsy.

Serum ACE levels were only reported in half of patients, with only 30% being elevated (25% of communicating and 33% of noncommunicating). All patients with communicating hydrocephalus in whom ESR was reported had normal values, while it was elevated in both patients with noncommunicating pathology in which it was reported. The most common abnormalities reported in CSF analysis include pleocytosis (100% of communicating and 63% of noncommunicating hydrocephalus) with a lymphocytic predominance in 75% of cases, and elevated protein (71% and 75% respectively). While the number of patients reported is low, the above findings suggest that serum ACE levels have limited sensitivity, but the specificity of the test might still make it valuable. Sarcoidosis should be a diagnostic consideration in patients with hydrocephalus with CSF pleocytosis or elevated protein levels in whom microbiological and cytology studies are negative.

While there has been no controlled trial for medical treatment of neurosarcoidosis, the consensus remains that corticosteroid therapy is first‐line. Ventriculoperitoneal (VP) shunt placement should be considered for symptomatic hydrocephalus.^8^ Of the cases reported in the literature, only one case of communicating hydrocephalus in neurosarcoidosis was successfully treated with corticosteroids alone, all other cases required surgical management (VP shunt, ventriculostomy, or ventricle fenestration) which in many cases took place before a definite diagnosis in order to manage the hydrocephalus. In the present case, VP shunt placement was done after three months of persistent and severe gait disturbance despite corticosteroid treatment, with rapid improvement in neurologic symptoms. Obviously, in cases of acute hydrocephalus, ventriculostomy can be life‐saving.

While most published reports of hydrocephalus in the context of sarcoidosis suggest a mortality rate as high as 75%, the great majority of those cases happened in patients with widespread disease that had not responded to treatment.^6^, ^8^, ^26^ In the current series of patients presenting with hydrocephalus, the reported mortality is 15%, with one patient dying suddenly from acute hydrocephalus, another from pulmonary embolism, and the last one from nosocomial pneumonia. The rest of the patients in whom outcomes were reported had either partial (35%) or complete (50%) recovery with treatment. Nevertheless, long‐term prognosis seems to depend more on the response of the underlying sarcoidosis to immunosuppressant treatment rather than the presence of hydrocephalus.

## 4 CONCLUSION

We present a rare case of neurosarcoidosis manifesting as subacute communicating hydrocephalus in a previously healthy patient. Though this pathology may pose a diagnostic challenge, it responds well to treatment and prognosis is favorable.

## CONFLICT OF INTEREST

None of the authors have a financial interest in any of the products, devices, or drugs mentioned in this manuscript. Rachel J. Saban, BS: None declared. Meaghan M. Berns, BA: None declared. Mazen M. Al‐Hakim, MD: None declared. Gustavo A. Patino, MD PhD: None declared.

## AUTHOR CONTRIBUTIONS

Rachel J. Saban, BS, Meaghan M. Berns, BA, Mazen Al‐Hakim, and MD Gustavo Patino, MD PhD: 1) substantially contributed to conception and design, or acquisition of data, or analysis and interpretation of data; 2) drafted and revised the article; 3) approved the final version; 4) involved in agreement to be accountable for all aspects of the work in ensuring that questions related to the accuracy or integrity of any part of the work are appropriately investigated and resolved.
